# Mechanisms in the Bed Nucleus of the Stria Terminalis Involved in Control of Autonomic and Neuroendocrine Functions: A Review

**DOI:** 10.2174/1570159X11311020002

**Published:** 2013-03

**Authors:** Carlos C Crestani, Fernando HF Alves, Felipe V Gomes, Leonardo BM Resstel, Fernando MA Correa, James P Herman

**Affiliations:** 1 Laboratory of Pharmacology, Department of Natural Active Principles and Toxicology, School of Pharmaceutical Sciences, São Paulo State University, UNESP, Araraquara, SP, 14801-902, Brazil; 2 Department of Pharmacology, School of Medicine of Ribeirão Preto, University of São Paulo, Ribeirão Preto, SP, 14049-900, Brazil; 3 Department of Psychiatry and Behavioral Neuroscience, University of Cincinnati, Metabolic Diseases Institute, Cincinnati, OH, 45237-0506, USA

**Keywords:** BNST, cardiovascular function, extended amygdala, neurotransmitters, HPA axis, vasopressin, stress and physical exercise.

## Abstract

The bed nucleus of the stria terminalis (BNST) is a heterogeneous and complex limbic forebrain structure, which plays an important role in controlling autonomic, neuroendocrine and behavioral responses. The BNST is thought to serve as a key relay connecting limbic forebrain structures to hypothalamic and brainstem regions associated with autonomic and neuroendocrine functions. Its control of physiological and behavioral activity is mediated by local action of numerous neurotransmitters. In the present review we discuss the role of the BNST in control of both autonomic and neuroendocrine function. A description of BNST control of cardiovascular and hypothalamus-pituitary-adrenal axisactivity at rest and during physiological challenges (stress and physical exercise) is presented. Moreover, evidence for modulation of hypothalamic magnocellular neurons activity is also discussed. We attempt to focus on the discussion of BNST neurochemical mechanisms. Therefore, the source and targets of neurochemical inputs to BNST subregions and their role in control of autonomic and neuroendocrine function is discussed in details.

## 1 INTRODUCTION

The bed nucleus of the stria terminalis (BNST) was defined originally by Johnston in 1923 as a gray matter structure that surrounds the stria terminalis and expands at its caudal and rostral ends [1]. The caudal end described by Johnston (1923) is now described as part of the amygdala. The rostral region, lying ventral to the lateral septal area and dorsal to the hypothalamic preoptic area, is referred to as the BNST. Johnston (1923) also noted that the BNST forms a continuum with amygdaloid structures [1], a view subsequently expanded by numerous authors [2-5]. Later studies led to identification of two major subdivisions of this continuum: a medial division that comprises the medial amygdaloid nucleus (MeA) and the medial division of the BNST, and a central division that is characterized by connections between the central amygdaloid nucleus (CeA) and the lateral BNST [3, 6]. Both subdivisions are connected by columns of cells that travel *via *the stria terminalis and the sublenticular part of the basal forebrain (ventral pathway) [3, 4, 6]. The idea that the BNST forms a continuum with the centromedial amygdala is further supported by a series of studies that note striking morphological and neurochemical similarities between these structures [7]. This continuum formed by the BNST and the amygdala gave rise to the notion of an “extended amygdala”, a concept introduced and championed by Lennart Heimer and colleagues [6]. Several excellent reviews have discussed in details the connections between the amygdala and BNST and the concept of extended amygdala [2, 4-6, 8].

Based mainly on innervation by neurons from the amygdala, the BNST was initially divided into lateral and medial divisions [9]. However, developmental and cyto- and chemo-architectonic studies led to an alternative designation of BNST subnuclei along an anterior to posterior gradient [10-12]. The anterior-posterior division of the BNST is also supported by functional experiments, noted below. The anterior division was further divided into anterodorsal, anterolateral and anteroventral areas [11, 12]. A subcommissural zone in the BNST anterior division was also identified [11], which based on connectional evidence was later assigned to the lateral region [5]. In all, twenty distinct cell groups are know identified within anterior and posterior divisions of the BNST [5]. A diagrammatic representation of coronal sections of the rat brain showing the anterior and posterior divisions of the BNST is presented in the Fig. (**1**).

The BNST is characterized by its connections with other limbic structures, as well as with hypothalamic and brainstem nuclei [13-18]. It plays an important role in control of autonomic, neuroendocrine and behavioral responses [19, 20]. It has reciprocal connections with the centromedial amygdala and receives projections from the hippocampus and medial prefrontal cortex (MPFC) [5, 16-18, 21, 22]. The BNST is thought to serve as a relay center between limbic cognitive centers and nuclei involved in processing of reward, stress and anxiety. Moreover, it receives ascending informationregarding systemic stressors, such as hypertension and hemorrhage [23, 24]. Therefore, the BNST seems to play an important role in the integration of physiological and behavioral responses,connecting structures such as amygdala, hippocampus and MPFC to hypothalamic and brainstem regions associated with autonomic and neuroendocrine functions [16, 20]. In addition to neuroanatomical evidence, this idea is supported by functional results demonstrating that BNST ablation inhibits cardiovascular and neuroendocrine responses elicited by amygdala and hippocampus stimulation [25-27].

It has been proposed that neurogenic mechanisms are involved in the physiopathology of several disorders, including cardiovascular pathologies and psychiatric disorders [28-31]. Although some clinical and preclinical studies propose a role of the BNST in the physiopathology of cardiovascular and psychiatric pathologies [32, 33], a detailed description of its role in control of physiological function is missing.In this review,we discuss the role of the BNST in modulation of autonomic and neuroendocrine activity. A description of BNST control of autonomic and neuroendocrine activity at rest and during emotional stress and physical exercise is presented. It has been documented the presence of a variety of neurotransmitters and neuro-modulators within the BNST, including inhibitory and excitatory amino acids, monoamines, acetylcholine, neuropeptides, nitric oxide and the endocannabinoid system [34-41]. Therefore, the present review attempts to focus on the discussion of the role of these BNST neurochemical mechanisms.The first section discusses some characteristics of neurochemical inputs to the BNST, such as source and targets of the terminals in the BNST subregions, as well as their role in control of autonomic function. The second section reviews evidence of a BNST involvement in modulation of neuroendocrine activity. 

## 2 BNST AND AUTONOMIC FUNCTION

Numerous studies have demonstrated a role of the BNST in control of cardiovascular function.Initial evidence was provided by electrical stimulation of the BNST [42-44]. Dunn & Williams (1995) demonstrated that BNST electrical stimulation elicited either depressor or pressor responses in anesthetized rats [42]. These contradictory results were explained by differences in the BNST region where the stimulation was done. The authors observed that stimulation of BNST medial division increased arterial pressure, stimulation of the lateral division decreased arterial pressure, whereas both pressor and depressor responses were observed following stimulation of the ventral region of the BNST. These pioneering results demonstrated that distinct subregions of the BNST might differently modulate the cardiovascular function. The same group observed that baroreceptor denervation did not affect changes of arterial pressure following BNST electrical stimulation [43], suggesting that cardiovascular changes evoked by BNST stimulation are not modulated by a baroreflex stimulation.

Electrical and chemical stimulation studies indicate that the anterior division is the critical BNST region involved in autonomic control [45-47]. Indeed, the largest cardiovascular responses are elicited more often when the anterior region of the BNST is stimulated [45, 47]. Neuroanatomical studies give support to a critical role of the anterior region in autonomic control. The anterior division (mainly anterolateral area) is the preferential region of the BNST connected with hypothalamic and lower brainstem regions associated with autonomic activity [5, 14]. In contrast, structural and functional data suggest that the posterior division is involved in controlling neuroendocrine and social behavior (defensive and reproductive) [5, 15, 48].

Whereas stimulation of the anterior BNST elicits cardiovascular changes, there does not appear to be a tonic influence of the BNST on maintenance of cardiovascular function [49-55]. Indeed, it was demonstrated that bilateral nonselective synaptic blockade within the BNST did not affect basal parameters of either arterial pressure or heart rate (HR) [50-52, 56].

The baroreflex is an important mechanism for moment-to-moment regulation of cardiovascular parameters [57], and dysregulation of this reflex mechanism is involved in several cardiovascular pathologies [58, 59]. Although BNST does not play a key role in maintenance of cardiovascular function, it is an important forebrain structure involved in tonic control of baroreflex activity. Sustained hypertension or hypotension caused by intravenous infusion of vasoactive drugs increased c-fos expression, a marker of neuronal activation, in the BNST [23], which is reduced following baroreceptor denervation [24]. In addition, BNST c-fos expression was also evoked by electrical stimulation of the aortic depressor nerve in rats [60]. We further reported that reversible ablation of BNST synapses enhanced bradycardiac response following blood pressure increases in unanesthetized rats, without affecting tachycardiac response to blood pressure decreases [50]. These results suggested that the BNST has a tonic inhibitory influence on bradycardiac component of the baroreflex. However, an opposite effect on baroreflex function following BSNT inhibition was recently reported [54]. It was observed that acute ablation of the BNST decreased the reflex bradycardia in anesthetized rats. Tachycardiac response during baroreceptor unloading was not investigated. It is well established that anesthesia buffer baroreflex function [61, 62]. Therefore, the anesthesia may explain, at least in part, the differences between our findings and those obtained in anesthetized animals. The role of the BNST in cardiovascular responses to chemoreflex activation was also investigated in a recent study [52], but BNST ablation did not affect either the pressor or bradycardiac response induced by intravenous infusion of potassium cyanide (KCN).

Some recent results have also suggested that the BNST play a key role in the integration of cardiovascular responses to emotional stress and physical exercise. Early evidence of BNST involvement in responses to stress was provided by demonstration that its stimulation produces behavioral consequences similar to those induced by restraint stress [63]. It was later demonstrated that local BNST neuro-transmission inhibition enhanced the HR increase associated with acute restraint stress without affecting the blood pressure increase [56]. A completely opposite result was observed in a conditioned stress model [64]. In turn, BNST ablation attenuated the freezing behavior and the increase in both blood pressure and HR induced by contextual fear conditioning [64]. Therefore, the role of the BNST in neurobiological mechanisms of emotional stress seems to depend on the type of stress. The influence of the BNST on cardiovascular responses to physical exercise was recently evidenced by demonstration that synaptic blockade of the BNST decreased both the pressor and tachycardiac response evoked by dynamic exercise on a rodent treadmill [51].

The involvement of several local neurotransmitters within the BNST in control of cardiovascular function at rest and during emotional stress and physical exercisehas been described. Below we describe the role of these local neurotransmitters in control of cardiovascular function by the BNST. Table **1** summarizes main findings and possible local and peripheral mechanisms associated with BNST control of cardiovascular activity.

### 2.1 Glutamate

Glutamate exerts its effects by interacting with metabotropic and ionotropic receptors [65]. Metabotropic glutamate receptors (mGluR) are divided into three groups: type I (mGluR 1 and 5), type II (mGluR 2, 3) and type III (mGluR 4, 6, 7 and 8) [66]. The ionotropic receptors are classified as N-Methyl-D-aspartate (NMDA) and non-NMDA receptors [67]. The non-NMDA receptors are further divided into α-amino-3-hydroxy-5-methyl-4-isoxazolepropionic acid receptor(AMPA) and kainite [67]. 

Functional and electrophysiological studies have suggested the presence of glutamatergic terminals in the BNST [68-72]. Source of glutamatergic innervation to the BNST is not completely established. However, available biochemical and electrophysiological results suggest that glutamatergic inputs to the BNST originate from neurons located in the ventral subiculum of the hippocampus and the infralimbic region (IL) of the MPFC [41, 73]. Based mainly on innervation of the BNST by neurons from the IL cortex and the ventral subiculum [22, 74], it has been suggested that glutamatergic neurons innervate the entire rostro-caudal extension of the BNST [48, 70].

Several studies have demonstrated that local BNST glutamatergic neurotransmission is an important neuro-chemical mechanism involved in modulation of cardiovascular function. Gelsema and Calaresu (1987) were the first to describe that the glutamatergic system present in the BNST is involved in control of cardiovascular function [75]. They demonstrated that microinjection of DL–homo-cysteate, a glutamate analog, into the BNST of urethane-anesthetized rats resulted in cardiovascular changes. The range of responses included both pressor and depressor effects on blood pressure and fall or no changes in HR, all of which depended on the site of stimulation within the BNST [75]. Indeed, results published later by Dunn & Williams (1995) gave support to the idea that glutamate action in distinct BNST regions may differently modulate cardiovascular function [42]. In urethane-anesthetized rats, they noted that glutamate microinjected into the medial portion of BNST anterior division caused increases on blood pressure and HR, stimulation of the lateral division decreased cardiovascular parameters, and both pressor and depressor response were observed when injection were centered into the BNST ventral region. Although the above results suggest a region-specific control of cardiovascular function by the BNST, glutamate microinjected into the BNST of conscious rats evoked exclusively depressor responses [46]. In fact, results suggesting that glutamate action in distinct BNST regions differently modulate cardiovascular function are controversial. Some groups have reported in anesthetized rats that stimulation of BNST with glutamate elicits depressor response regardless of region stimulated [45, 55, 76].

The mechanisms involved in depressor and bradycardiac responses to glutamate microinjection into the BNST have been extensively investigated. The largest cardiovascular responses are elicited more often when glutamate microinjection sites are centered into the anterior BNST [45, 75], consistent with the rostral division being critical in cardiovascular control. In unanesthetized rats, the fall in blood pressure mediated by anterior BNST stimulation was accompanied by an increase in the conductance of vessels supplying the hindlimb musculature without changes in conductance in renal and mesenteric beds [46]. Also, the decrease in both blood pressure and HR induced by glutamate microinjection into the BNST of chloralose-anesthetized rats were not affected by intravenous treatment with a muscarinic receptor antagonist, but were abolished after ganglion blockade [45]. Together, above results suggest that decrease of blood pressure and HR evoked by glutamate administration into the anterior BNST are mediated by an inhibition of sympathetic fibers to the vasculature supplying the hindlimb and the heart [45, 76]. 

The local glutamatergic mechanisms and neural pathways involved in control of cardiovascular function by BNST glutamatergic neurotransmission are not completely understood. A recent study reported that fall in blood pressure and HR evoked by glutamate microinjection into the BNST of anesthetized rats were reduced by local BNST pretreatment with either a selective NMDA or non-NMDA glutamate receptor antagonist [55]. In addition, glutamate cardiovascular responses were inhibited after nonselective synaptic ablation of the caudal ventrolateral medulla (CVLM) [76]. The CVLM projects to and inhibits sympathetic premotor neurons in the rostral ventrolateral medulla, thus decreasing sympathetic preganglionic neuronal outflow [77]. Therefore, above evidence suggest that depressor and bradycardiac responses to glutamate microinjected into the BNST are mediated by co-activation of local NMDA and non-NMDA glutamate receptors that, in turn, stimulate a pathway originating in the BNST and relaying in the CVLM.

Glutamatergic mechanisms within the BNST play a key role in cardiac baroreflex responses. Bilateral microinjection of a selective NMDA antagonist, but not a selective non-NMDA antagonist, into the BNST increased bradycardiac responses to blood pressure increments [72]. Considering the aforementioned results showing that nonselective BNST ablation enhance reflex bradycardia [50], it is possible that the inhibitory influence of the BNST on reflex bradycardia is mediated by activation of local NMDA glutamate receptors. 

Microdialysis results demonstrate absence of a glutamatergic tonic activity in the BNST [70]. Indeed, either unilateral [55] or bilateral [72] treatment of the BNST with glutamate receptor antagonists does not induce any significant change in baseline values of both arterial pressure and HR. 

### 2.2 Gamma-aminobutyric Acid

There are two main classes of gamma-aminobutyric acid (GABA) receptors: GABA_A_ and GABA_B_ [78, 79]. GABA_A _receptors are pentameric ligand-gated ion channels (chloride channels) that mediate the majority of fast inhibitory neurotransmission in the central nervous system [79]. In contrast, GABA_B_ receptors are metabotropic receptors that act by coupling to G(i)/G(o) protein and mediate slow GABA effects [78]. 

The activity of BNST neurons is regulated by GABAergic inputs from both intrinsic and extrinsic sources. The vast majority of MeA and CeA neurons are positive for GABA and glutamic acid decarboxylase (GAD), the enzyme that converts glutamate to GABA and is responsible for the majority of de novo GABA synthesis [80, 81]. Based on these results, it has been proposed that extrinsic projections of these amygdalar nuclei are predominantly GABAergic [81]. Neuroanatomical results have indicated that the CeA densely innervate the BNST anterior division, whereas MeA neurons project preferentially to the posterior division [5]. These available anatomical and neurochemical results suggest that GABAergic inputs from amygdala innervate the entire rostro-caudal extension of the BNST. 

In addition to extrinsic sources, a network of internal GABAergic neurons within the BNST has been described. This idea is supported by observation that the majority of BNST neurons are GABAergic [74, 80]. Moreover, electro-physiological data suggest the presence of a functional GABAergic local circuit [69]. However, additional studies are necessary for better characterization of a possible network of local GABAergic interneurons. 

A limited number of studies have investigated the role of BNST GABAergic neurotransmission in control of cardiovascular function. A recent study demonstrated that microinjection of the selective GABA_A_ receptor antagonist bicuculline methiodide (BMI) into the BNST significantly increased both blood pressure and HR in anesthetized rats [82]. However, microinjection of muscimol, a GABA_A_ receptor agonist, or the selective GABA_B _receptor antagonist phaclofen did not produce any significant cardiovascular changes. The authors also observed that BMI responses were not affected by intravenous pretreatment with a muscarinic receptor blocker. In contrast, a ganglion blocker inhibited the HR response without affecting the blood pressure change, whereas a selective V_1_-vasopressin receptor antagonist inhibited exclusively the pressor response [82]. These results demonstrated that BNST GABAergic neurotransmission, *via *activation of local GABA_A_ receptor, play a tonic role on maintenance of blood pressure and heart rate. The pressor and tachycardiac responses were mediated by a modulation of vasopressin release into the circulation and cardiac sympathetic activity, respectively [82]. Also, orexin-containing neurons in the medial hypothalamus seems to be part of the neural pathway involved in control of cardiorespiratory function by GABA receptors within the BNST [47]. The tonic modulation of cardiovascular function by GABAergic mechanisms in the BSNT is consistent with electrophysiological results suggesting that BNST neurons are tonically inhibited by GABA receptors [69]. 

The influence of chronic loss of inhibitory GABAergic tone in the BNST in panic-like responses was investigated [83]. However, the baseline cardiovascular parameters were not reported. Moreover, GABA inhibition in the BNST did not affect tachycardia, hypertension and tachypnea induced by sodium lactate infusions [83]. 

### 2.3 Noradrenaline

Adrenoceptors were initially subdivided in two subtypes: α and β [84]. Further, α-adrenoceptor was subdivided in two subtypes (α_1_ and α_2_) and three subtypes of β were described (β_1_, β_2_, and β_3_) [85-87]. Currently, nine adrenoceptors subtype have been characterized, including three subtypes of α_1_ (α_1A_, α_1B_, α_1D_) and α_2_ (α_2A_, α_2B_, α_2C_) adrenoceptors [85, 87, 88]. 

Among the numerous neural inputs to the BNST, noradrenergic synaptic terminals are among the prominent [34, 37]. In fact, the BNST is one of the major targets of noradrenergic innervation in the brain [36, 89, 90]. Nonetheless, the distribution of noradrenergic terminals in the BNST is heterogeneous. The highest noradrenaline levels are found in anterior division of the BNST [91]. Moreover, noradrenergic terminals are mainly located in the ventral portion of the BNST [34, 37]. Noradrenergic neural terminals in the BNST originate mainly from neurons located in the A1, A2 and A5 brainstem nuclei, and in a lesser degree from the A6 [92-96]. Moreover, projections from the A1 noradrenergic nucleus to the BNST send collateral projections to the CeA and the PVN [97, 98]. Importantly, electrophysiological studies have demonstrated that noradrenaline play an inhibitory role in activity of BNST neurons [63, 99, 100].

**Fig. (2):**
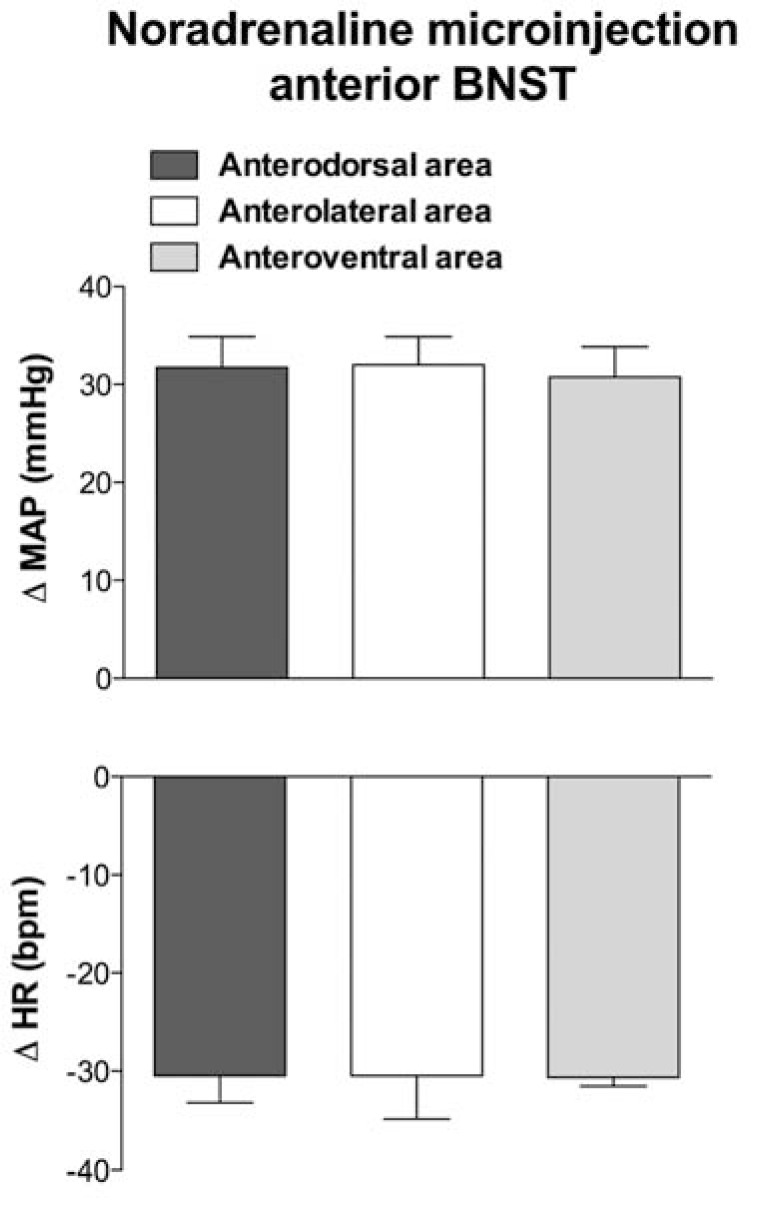
Changes in mean arterial pressure (Δ MAP) and heart rate (ΔHR) in response to noradrenaline (10nmol/100nL) microinjection into the anterodorsal, anterolateral and anteroventral area in the anterion division of the BNST. Columns represent the mean and bars the SEM. Cardiovascular changes were not different when noradrenaline was microinjected into the different regions of BNST anterior division (One-way ANOVA, *P*>0.05).

Noradrenergic cell groups in medullary regions involved in control of cardiovascular function are important source of BNST noradrenergic innervation [93, 95]. Functional studies further indicate that local noradrenaline microinjection into the BNST evokes cardiovascular responses. Noradrenaline microinjection into the BNST of unanesthetized rats increases arterial pressure and causes bradycardia [101]. Cardiovascular changes were observed only when noradrenaline was microinjected into the anterior division of the BNST (our laboratory, data not published), consistent with results that noradrenergic innervation are mainly located in this region of the nucleus [34, 37, 91, 101]. Also, cardiovascular changes were not different when noradrenaline was microinjected into the anterolateral, anterodorsal or anteroventral regions of the anterior BNST (Fig. **2**). Cardiovascular responses to noradrenaline microinjected into the BNST were blocked by intravenous pretreatment with a selective V_1_-vasopressin receptor antagonist [101], suggesting its mediation by vasopressin release into circulation. Detailed discussion of the BNST role in control of vasopressin release into the circulation is presented below (BNST and neuroendocrine function). 

The pressor and bradycardiac responses evoked by noradrenaline microinjected into the BNST was only partially reduced after local treatment with either a selective α_1_- or α_2_-adrenoceptor antagonist [102]. The response was completely abolished only when both α_1_- and α_2_-adrenoceptor antagonists were combined, thus indicating that co-activation of α_1_- and α_2_-adrenoceptor within the BNST is required for noradrenergic modulation of cardiovascular function [102]. In addition, BNST pretreatment with a selective β_1_-adrenoceptor antagonist, but not a β_2_-adrenoceptor antagonist, enhanced cardiovascular responses to noradrenaline [102], indicating that activation of local β_1_-adrenoceptors opposes actions at the α-adrenoceptors. 

Involvement of local noradrenergic neurotransmission in control of BNST baroreflex function was also demonstrated. Crestani and collaborators (2008) reported that bilateral microinjection of a selective α_1_-adrenoceptor antagonist increased the gain of reflex bradycardia in response to blood pressure increases [103]. Effects on baroreflex activity were no longer observed when animals were pretreated intravenously with a cholinergic muscarinic receptor antagonist. These results provided evidence that local α_1_-adrenoceptors are involved in tonic BNST regulation of the baroreflex bradycardiac response controlling cardiac parasympathetic activity. Bilateral microinjection of either selective α_2_- or β-adrenoceptor antagonists into the BNST did not affect baroreflex activity [103]. 

Local adrenoceptors also mediate control of stress- and exercise-evoked cardiovascular adjustments at the BNST. It was initially demonstrated that BNST pretreatment with a selective α_1_-adrenoceptor antagonist enhanced the HR increase associated with acute restraint stress without affecting pressor responses [56]. This effect was similar to that observed after nonselective blockade of BNST synapses [56], indicating an involvement of local noradrenergic neurotransmission in inhibition of restraint-related HR responses. Bilateral microinjection into the BNST of either a selective α_2_- or β-adrenoceptor antagonist did not affect restraint-evoked cardiovascular responses. A completely opposite influence of BNST noradrenergic neurotransmission was observed in a conditioned stress model [53]. Indeed, BNST pretreatment with either a selective α_1_- or β_1_-adrenoceptor reduced both freezing and autonomic responses induced by an aversive context, observed in the contextual fear-conditioning model [53]. These results corroborate data obtained after nonselective ablation,indicating that BNST differentially modulates cardiovascular responses to unconditioned and conditioned stress [56, 64], and reinforce the idea that the role of the BNST in neurobiological mechanisms of emotional stress depends on the type of stress.

The role of BNST noradrenergic neurotransmission in autonomic control during exercise was suggested by a recent study from our group demonstrating that local α_1_- and α_2_-adrenoceptors differentially modulate cardiovascular responses to a protocol of dynamic exercise [49]. Bilateral microinjection of a selective α_1_-adrenoceptor antagonist into the BNST enhanced the HR increase evoked by running on a rodent treadmill, without affecting the blood pressure increase.In contrast, BNST pretreatment with a selective α_2_-adrenoceptor antagonist reduced exercise-evoked pressor response without changing the tachycardiac response [49]. These results brought the first direct evidence for the involvement of brain adrenoceptors in cardiovascular responses observed during dynamic exercise. Considering that blockade of α_2_-adrenoceptor and the nonselective synaptic ablation within the BNST produced similar effects on exercise-evoked pressor response [51], it is possible that local α_2_-adrenoceptors mediate, at least in part, a BNST influence on blood pressure during exercise. The neurotransmitter(s) in the BNST that are involved in its influence on tachycardiac response to exercise remain to be elicited.However, the facilitation of exercise-evoked tachycardiac response following the blockade of α_1_-adrenoceptor within the BNST indicates that activation of these receptors may have a physiological role in counteracting excessive cardiac activation during dynamic exercise [49]. 

*In vivo* microdialysis results demonstrated a noradrenergic tonic activity in the BNST [104]. However, either unilateral [102] or bilateral [49, 53, 56, 103] treatment of the BNST with adrenoceptor antagonists did not induce any significant change in baseline values of either blood pressure or HR. Therefore, apart of a key physiological role played by BNST adrenoceptors in control of baroreflex activity and cardiovascular responses to stress and physical exercise [49, 53, 56, 103], BNST noradrenergic neurotransmission does not play a role in tonic maintenance of cardiovascular function.

### 2.4 Acetylcholine 

There are two main classes of acetylcholine receptors: muscarinic and nicotinic receptors [105-107]. The muscarinic receptors are metabotropic receptors [105], and mediate physiological effects *via *five muscarinic receptor subtypes(M_1, _M_2_, M_3_, M_4_ and M_5_) [105, 106]. Nicotinic receptors are ionotropic composed of five subunits arranged symmetrically around a central ion-conducting pore [107]. 

The activity of neurons within the BNST is modulated by local acetylcholine release. Indeed, cholinergic terminals were identified in the BNST [38]. Moreover, autoradiographic ligand-binding studies identified the presence of both muscarinic and nicotinic cholinergic receptors in the BNST [108, 109]. Neuroanatomical and electrophysiological evidence have suggested the presence of cholinergic terminals throughout the BNST [38, 63, 110]. Moreover, acetylcholine seems to play an excitatory role in activity of BNST neurons [63, 110]. However, although some data indicate that cholinergic innervation to BNST is supplied by the latero-dorsal tegmental nucleus [111], the source of these inputs is not completely established.

Few studies have investigated the involvement of BNST cholinergic neurotransmission on cardiovascular modulation. We have observed that microinjection of carbachol, a nonselective muscarinic receptor agonist, into the BNST of unanesthetized rats caused increase in blood pressure that was followed by bradycardia [112]. These responses were abolished after local pretreatment with a selective M_2_ receptor antagonist as well as after intravenous treatment with a selective V_1_-vasopressin receptor antagonist [112]. Therefore, these results suggested that cardiovascular effects of carbachol were mediated by activation of local M_2_ receptors that, in turn, evokes acute release of vasopressin into the systemic circulation. Detailed discussion of the BNST role in control of vasopressin release into the circulation is presented below (BNST and neuroendocrine function).

A recent study demonstrated that microinjection into the BNST of the endogenous neurotransmitter acetylcholine also caused pressor responses, but without changing HR in urethane-anesthetized rats, and effects were blocked by local pretreatment with a nonselective muscarinic receptor antagonist [54]. Urethane decreases the baroreflex control of HR [62]. Although authors reported reflex responses in response to intravenous administration of phenylephrine, it remains possible that anesthesia can account for the absence of bradycardia following acetylcholine microinjection into the BNST of anesthetized animals. Other interesting difference between cardiovascular responses following local administration of carbachol or the endogenous neurotransmitter acetylcholine was that although acetylcholine microinjection into the BNST evokes pressor response in anesthetized animals, urethane-anesthesia inhibited carbachol cardiovascular responses [112]. The reasons for the discrepancy are not clear. Therefore, further studies are necessary to clarify whether cardiovascular responses to carbachol microinjection into the BNST mimic responses evoked by local treatment with the endogenous neurotransmitter.

Nasimi & Hatam (2011) also investigated a possible influence of BNST cholinergic neurotransmission in baroreflex control of the HR [54]. They observed that although bilateral treatment of the BNST with a muscarinic receptor antagonist did not affect reflex bradycardia, local acetylcholine microinjection significantly reduced bradycardiac baroreflex response in urethane-anesthetized rats [54].

### 2.5 Nitric Oxide

Nitric oxide (NO) is a small/short-lived molecule that modulates numerous physiological functions [113]. NO was initially described as the endothelium-derived relaxing factor (EDRF) [114, 115], been previously described by Furchgott and Zawadzki (1980) [116]. However, a set of key findings led to the identification of NO as a signalling molecule in the brain and other tissues (for review see [117]).

NO is synthesized from l-arginine by three cell-specific nitric oxide synthases (NOS) isoforms. Neuronal nitric oxide synthase (nNOS or Type I) and endothelial nitric oxide synthase (eNOS or Type III) are constitutively expressed enzymes, whose activities are stimulated by increases in intracellular Ca^+2^, whereas inducible nitric oxide synthase (iNOS or Type II) is calcium-independent and mediates immune functions of NO [118]. Although other mechanisms have been described (e.g., nitration of protein thiol groups or S-nitration and inhibition of mitochondrial cytochrome c oxidase) [117], the majority of NO effects are mediated by activation of soluble guanylyl cyclase(sGC) and consequent cyclic guanosine monophosphate(cGMP) formation [117, 119].

The nNOS has been demonstrated as the major isoform involved on NO synthesis in the central nervous system [120]. Garthwaite and collaborators demonstrated that activation of NMDA glutamate receptors present in postsynaptic neurons in the central nervous system results in the formation of NO, through a mechanism dependent on Ca^+2^ [121]. Therefore, it has been proposed that the nNOS enzyme in the brain is activated in response to Ca^+2^ influx following the activation of NMDA receptors by glutamate [117, 122]. NO has been demonstrated to play an important role in several cerebral functions and/or dysfunctions, including regulation of neuronal excitability, synaptic plasticity (e.g., long-term potentiation and depression), neurotoxicity, neuroprotection, modulation of several behaviors and, importantly, control of cardiovascular function [117, 119].

NOS-positive neurons were identified in numerous regions associated with control of cardiovascular function, including the BNST [40]. However, a participation of BNST nitrergic mechanisms in control of cardiovascular function has not been extensively investigated. To address this issue, a recent study from our group demonstrated that bilateral microinjection of either the nonselective NOS inhibitor L-NG-Nitroarginine methyl ester (L-NAME) or a selective nNOS inhibitor into the BNST increased bradycardiac baroreflex response [72]. Also, BNST treatment with a NO scavenger caused an effect similar to that observed after NOS blockade [72]. Interestingly, changes on baroreflex activity following local NOS blockade or NO scavenger was similar to that observed after BNST treatment with a selective NMDA receptor antagonist, suggesting a NMDA receptor–NO interaction [72]. This idea was further reinforced by observation that changes on reflex bradycardia induced by local NMDA receptors were reverted by BNST treatment with a NO-donor [72].

Further studies are required for full characterization of cardiovascular control by BNST NMDA receptor-NO pathway. Moreover, the expression of iNOS and eNOS has been documented in brain cells [123, 124], suggesting a role of other NOS isoforms in control of NO function in the central nervous system. In addition, signaling from cerebral vascular beds to neurons by endothelial production of NO has been proposed [117]. Therefore, investigation of mechanisms other than those involving nNOS is also necessary. 

### 2.6 Cannabinoids

The neuronal endocannabinoid system is classically described as being composed of two best-characterized endocannabinoids: anandamide and 2-arachidonoylglycerol [125]. Endocannabinoid signalling is mediated by the cannabinoid receptors type-1 (CB1) and type-2 (CB2), as well as by proteins responsible for endocannabinoid synthesis, transport and degradation [125, 126]. 

The endocannabinoid system plays an important regulatory role in several brain functions. The CB1 receptor is highly expressed throughout the central nervous system and modulates synaptic transmission and plasticity in many brain regions, including the BNST [35, 127]. Activation of these receptors on axon terminals regulates ion channel activity, inhibiting neurotransmitter release [125, 128]. The CB1 receptor is present in glutamatergic and GABAergic terminals in the BNST [127, 129, 130]. Moreover, electro-physiological studies have demonstrated that activation of CB1 receptors inhibits inhibitory and excitatory transmission in BNST slices [73, 127], which could be important for the regulation of behavioral and physiological responses in this area. Despite these pieces of evidence, no study so far has investigated the effects of direct injections into the BNST of CB1 receptor agonist or antagonist on cardiovascular function.

A recent study from our group have shown that systemic treatment with cannabidiol, a non-psychotomimetic component of *Cannabis sativa* that has shown anxiolytic properties, was able to reduce c-Fos expression in the BNST following re-exposure to an aversive context previously paired with footshocks [131]. These results provide evidence that the BNST is involved in antiaversive effects of cannabidiol. We have also shown that direct injection of cannabidiol into the BNST attenuated the behavioral and cardiovascular changes induced by contextual fear conditioning [132]. In addition, BNST treatment with cannabidiol enhanced tachycardiac response evoked by acute exposure to restraint stress, without affecting blood pressure changes [133]. However, cannabidiol into the BNST did not induce any significant change in baseline values of arterial pressure and HR. Therefore, it is unlikely that the attenuation of the cardiovascular responses to aversive context depends on direct cardiovascular effects, but rather on an attenuation of the emotional response. In agreement with this proposal, intra-BNST administration of cannabidiol has been shown to induce anxiolytic-like effects in animal submitted to elevated plus-maze and Vogel conflict test [134]. However, a direct influence of cannabidiol in cardiovascular function could not be ruled out. Accordingly, we have recently documented that cannabidiol microinjected into the BNST facilitates bradycardiac baroreflex response [135]. 

The mechanisms associated with effects of cannabidiol are still poorly understood. Although cannabidiol has low affinity for cannabinoids receptors [136], it can block the reuptake of the endogenous cannabinoid anandamide and its metabolism by the enzyme fatty acid amide hydrolase [137, 138]. Also, cannabidiol can activate TRPV_1_ receptors [137], enhance adenosine signaling through inhibition of uptake [139], and modulate serotoninergic neurotransmission by allosterically inhibiting 5-HT_3_ receptor [140] or acting as agonist at the 5-HT_1A_ receptor [141]. 

The BNST receives dense serotoninergic innervation from caudal regions of the dorsal raphe nucleus [142, 143]. Moreover, BNST neurons express a variety of serotonin receptors, and 5-HT_1A_ receptors is one the most prevalent receptor subtypes expressed [144]. In this way, serotonin elicits inhibitory responses, evoking membrane hyper-polarization, in the majority of BNST neurons by activation of postsynaptic 5-HT_1A_ receptors [145, 146]. Interestingly, all effects following CBD microinjection into the BNST were inhibited after local pretreatment with a 5-HT_1A _antagonist [132, 133, 135]. Therefore, activation of local 5-HT_1A_ seems to be a key mechanism involved in CBD action in the BNST.

### 2.7 Corticotropin-releasing Factor

Corticotropin-releasing factor (CRF) was first isolated from ovine hypothalamus in 1981, and was initially characterized as a regulator of adrenocorticotropic (ACTH) hormone secretion from pituitary cells [147]. However, it was further demonstrated that effects of CRF extend beyond the action as a hormone and its role in control of cardiovascular and gastrointestinal function, behavioral and physiological responses to stress, food intake and satiety has been described [148-153]. The mammalian CRF system is composed by the CRF and other three CRF-like peptides denominated urocortin 1, urocortin 2 and urocortin 3 [154]. Physiological effects of CRF and the urocortins are mediated by two receptors, CRF_1_ and CRF_2_, and a CRF binding protein [154].

Populations of CRF-containing fibers have been found in the oval and fusiform region of the anterior BNST [155, 156]. Furthermore,high densities of CRF-containing terminals are found throughout rostrocaudal extent of the BNST [157], and CRFergic inputs arising from the amygdala seem to be confined in oval region [157]. Both CRF receptors are expressed in the BNST [158, 159]. 

It has been suggested that stress activates CRF system in the BNST [160, 161]. A role of BNST CRFergic neuro-transmission in behavioral response to aversive stimuli is well described [162, 163]. However, to our knowledge, only one study investigated the influence of this system in control of cardiovascular function. Nijsen and collaborators (2001) demonstrated that CRF administration into the BNST increased HR without affecting PQ interval of the electrocardiogram under resting conditions in conscious rats, thus suggesting co-activation of sympathetic and parasympathetic cardiac outflow [164]. They also observed that BNST pretreatment with a nonselective CRF receptor antagonist reduced the PQ increase and enhanced the tachycardia induced by conditioned contextual fear, thus suggesting that activation of local CRF receptors within the BNST during conditioned emotional responses inhibits cardiac vagal activity [164].

In summary, numerous results have demonstrated that BNST play a key role in control of cardiovascular function. Several local neurochemical mechanisms mediate the BSNT modulation of autonomic and cardiovascular function. Interestingly, some data indicate that cardiovascular regulation by some local neurotransmitters is mediated by neuroendocrine mechanisms (vasopressin release into the circulation), thus suggesting a coordinated control of cardiovascular and neuroendocrine function by the BNST. In the next session we discuss the role of the BNST in control of neuroendocrine function.

## 3 BNST AND NEUROENDOCRINE FUNCTION

Paraventricular (PVN) and supraoptic (SON) nuclei of the hypothalamus are final components of neuronal pathways that regulate a variety of neuroendocrine functions. The SON and PVN contain magnocellular neurons [165]. Vasopressin and oxytocin are synthetized by these magnocellular neurons and stored in the posterior pituitary for further release into the circulation [165, 166]. These peptides regulate water balance, cardiovascular function, parturition and lactation [167-171]. In addition to magnocellular cells, the PVN is also composed of parvocellular neurons [172]. Separate parvocellular neuronal populations were reported to project either to the median eminence or to autonomic targets in the brainstem and spinal cord [173]. 

In this section we discuss the role of the BNST in controlling the activity of the hypothalamus-pituitary-adrenal (HPA) axis and hypothalamic magnocellular neurons. Possible neural pathways culminating in the PVN and SON and neurochemical mechanisms involved in control of these neuroendocrine functions by the BNST are presented. 

### 3.1 BNST and Hypothalamus-pituitary-adrenal Axis

The BNST plays a major role in regulation of HPA responses to stress. Several of the subregions comprising the BNST, including anterolateral and posteromedial subdivisions, send direct projections to corticotropin releasing-hormone (CRH) containing parvocellular regions of the PVN [13, 15, 174], providing a mechanism for direct actions on HPA axis output. Anatomical studies indicate that the vast majority of BNST input to the parvocellular PVN is GABAergic [74], suggesting that BNST provides largely inhibitory input to HPA axis output neurons. Innervation by BNST GABA neurons may contribute to the pronounced inhibitory tone on parvocellular PVN neurons [16]. Indeed, lesions of some BNST subregions, most notably the posterior medial zone, increase basal CRH and parvocellular arginine vasopressin (AVP) mRNA expression, suggesting release of inhibition on ACTH secretagogue gene expression [175]. In addition, lesions of the posterior BNST result in elevated corticosterone release and increased parvocellular PVN Fos activation following acute restraint [48], indicating that this cell group participates in inhibition of responses to psychogenic stressors.

However, some regions of the BNST, most notably the fusiform nucleus, contain substantial populations of PVN-projecting CRH neurons [13]. Local PVN or icv injections of CRH cause parvocellular PVN Fos activation and induce corticosterone release, suggesting that at least some BNST neurons may participate in activation of the HPA axis. This possibility is supported by data demonstrating decreased stress-induced ACTH, corticosterone and PVN c-fos mRNA induction following lesions of the anteroventral (but not anterodorsal) CRH cell groups of the BNST [48].

The effects of the BNST on stress responsiveness are dependent upon the duration of exposure. For example, lesions of the anteroventral BNST reduce HPA axis response to acute stress, but markedly potentiate HPA stress responses following chronic unpredictable stress exposure [176], suggesting that different cell populations may be recruited by acute vs. chronic stress.Notably, lesions of the posterior medial BNST potentiate stress responses after chronic unpredictable stress [177], consistent with a role in limiting sensitization of the HPA axis after prolonged stress.

The anteroventral region of the BST expresses both GABA and CRH [13]. Immunolesioning experiments demonstrate that there are mutually exclusive populations of GABA and CRH neurons [17]. Moreover, selective damage of anteroventral BNST GABAergic (but not CRH) cells causes increases in stress-induced HPA axis activation and PVN Fos expression [17], suggesting functional segregation among intermingled BNST neurons in this region. Anteroventral BNST CRH neurons are activated by stimuli that cause HPA axis activation (e.g., interleukin 1-beta) [178], suggesting that activation of the HPA axis may be mediated, at least in part, by BNST CRH neurons.

The BNST receives rich innervation from limbic forebrain structures such as the ventral subiculum, medial amygdala and central amygdala [16], as well as more limited input from the medial prefrontal cortex [17, 18]. Neurons in the ventral subiculum, prelimbic cortex and medial amygdala project to PVN-projecting neurons in the posteromedial BNST, suggesting that the BNST serves as a relay between limbic processing of emotional information with eventual elaboration of an HPA axis stress response. Indeed, there is evidence for co-innervation of PVN-projecting BNST neurons by the ventral subiculum and prelimbic cortex [179], indicating that limbic information may be integrated at the level of the individual BNST neuron.

**Fig. (3):**
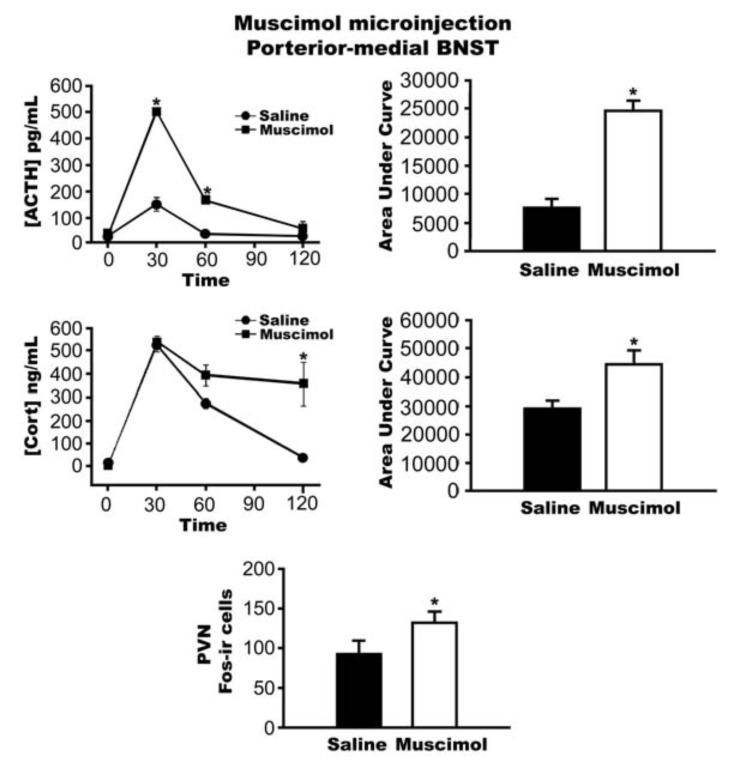
Plasma ACTH (*P*<0.05) and corticosterone (cort)(*P*<0.05) response to an acute 30 min restraint stress challenge was enhanced in animals that received the GABA-A receptor agonist muscimol into the posterior-medial BNST. Muscimol microinjection into the posterior-medial BNST also increased c-fos expression in the PVN of the hypothalamus(*P*<0.05). Data are shown as mean±SEM.*P<0.05 versus saline.

GABAergic neurons comprise the principle target of the primary amygdalar regions innervating the BNST (medial and central amygdaloid nuclei). Moreover, the vast majority of BNST neurons are GABAergic in phenotype (>90%) [74]. Consequently, activation of the HPA axis may be mediated in part by disinhibition of PVN-projecting BNST neurons (i.e., inhibition of an inhibitory neuron) [16]. To test this hypothesis, our group performed injections of muscimol, a GABA-A receptor agonist, into the posterior-medial BNST. Muscimol causes hyperpolarization of GABA-A receptor-bearing neurons and results in a net inactivation of neurons in the area of injection. Our data indicate that local inactivation of the posterior BNST enhances HPA responses to acute restraint stress (Fig. **3**), consistent with the hypothesized disinhibitory mechanism of action. These data are supported by immunolesion studies, which demonstrate that destruction of ventral BNST GABA neurons enhance HPA axis responses to acute stress [17].

**Fig. (4):**
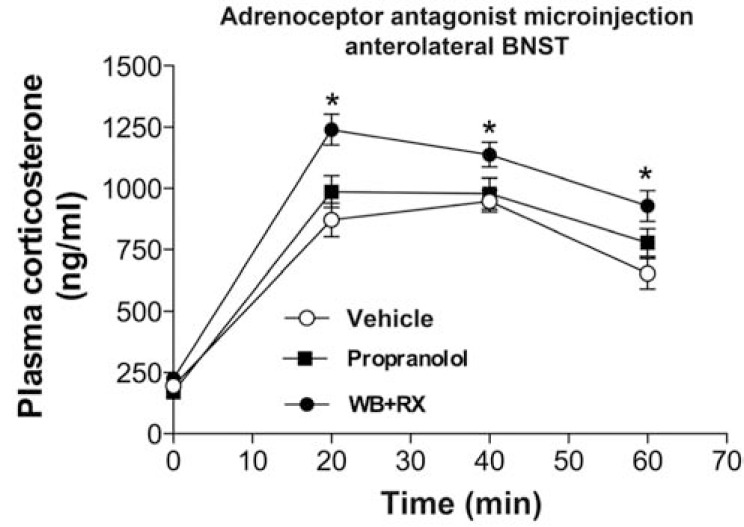
Plasma corticosterone response to an acute 30 min restraint stress challenge in animals that received bilateral microinjection into the anterolateral BNST of vehicle (100nL), the β-adrenoceptor antagonist propranolol (10nmol/100nL) or a cocktail containing the selective α_1_-adrenoceptor antagonist WB4101 (10nmol/100nL) and the selective α_2_-adrenoceptor antagonist RX821002 (10nmol/100nL) (WB+RX). The response in animals treated with the cocktail of WB+RX was enhanced (*P*<0.05), whereas propranolol microinjection did not affect plasma corticosterone response (*P*>0.05). Circles represent the mean and bars the SEM; n=12-14. *P<0.05 versus vehicle group, two-way ANOVA followed by Bonferroni’s *post hoc test*.

The BNST receives substantial input from brainstem noradrenergic neurons, with particularly dense projections to the anterolateral and anteroventral regions [70, 180]. The relationship between BNST noradrenergic innervation and motivated behaviors, e.g., addiction and relapse, is well characterized, and numerous studies indicate that stress exposure causes release of noradrenaline in the BNST. In general, BNST noradrenaline release causes inhibition of HPA axis responses to stress [70]. Similarly, recent data from our group indicate that microinjection of a cocktail of α_1_- and α_2_-adrenoceptor antagonist(WB4101 and RX821002) into the anterolateral BNST (but not the β-adrenoceptor antagonist propranolol) increases corticosterone release following acute restraint stress (Fig. **4**), further suggesting an inhibitory influence of BNST noradrenergic neuro-transmission on PVN neural activity.However, presynaptic facilitation of norepinephrine release by the α_2_-adrenoceptor antagonist yohimbine increases PVN Fos activation, an effect that can be attenuated by DBH-DSAP lesions of the BNST [181]. Noradrenergic effects appear to be mediated by a combination of presynaptic inhibition of noradrenaline release and inhibition of glutamate neurotransmission [182, 183].

Both estrogen (ERalpha, ERbeta) and androgen (AR) receptors are abundantly expressed in the BNST, particularly its posterior-medial subdivisions [184, 185]. A substantial number of AR neurons project to the PVN [186]. Recent data suggest that dihydrotestosterone implants into the posterior BNST enhance stress-induced PVN Fos activation and increase PVN AVP mRNA, suggesting that BNST AR activation may promote HPA axis activation [187]. The role of BST ERs remains to be evaluated.

The BNST contains numerous other peptidergic terminal fields and cell groups, expressing proenkephalin, nociceptin/orphanin FQ (N/OFQ), cholecystokinin, galanin, substance P, and pituitary adenylate cyclase-activating peptide (PACAP), as well as others [12, 188-190]. While not extensively studied, some data exist to suggest these at least some of these peptidergic neurons are important in HPA axis regulation. For example, local injection of N/OFQ increases HPA axis responses to novelty [191]. Electrophysiological data suggest that N/OFQ inhibits the majority of BNST neurons [192], suggesting that N/OFNQ activates the PVN *via *inhibition of inhibitory BNST output neurons. Microinjection of a galanin antagonist into the BNST increases stress-induced anxiety and ACTH responses [193], suggesting that BNST galanin receptors increases activation of the HPA axis in the context of stress. Other peptides resident in the BNST, such as PACAP, can directly activate CRH neurons [194], suggesting possible excitatory peptidergic projections to the PVN. Notably, BNST PACAP is up-regulated by chronic stress, a condition that causes hyperactivity of the HPA axis [195].

### 3.2 BNST and Hypothalamic Magnocellular Neurons

Vasopressin and oxytocin have opposing actions in cardiovascular control: vasopressin is a potent vasoconstrictor and increase blood pressure *in vivo*, whereas oxytocin produces vasodilatation [168, 170, 196, 197]. As previously mentioned, pressor responses following local microinjection of noradrenaline, carbachol and GABA into the BNST appear to be mediated by an acute release of vasopressin into the circulation [82, 101, 112]. Evidence of the existence of massive neural projection from the BST to magnocellular neurons in the PVN and SON provides neuroanatomical support for a BST influence in activity of magnocellular vasopressinergic neurons [14]. Indeed, pressor response to noradrenaline microinjection into the BNST was blocked after blockade of local PVN synapses, but not after SON inhibition [198]. Moreover, pretreatment of the PVN with a selective non-NMDA glutamate receptor antagonist, but not with a selective NMDA glutamate receptor antagonist, also inhibited cardiovascular responses to activation of adrenoceptors into the BNST [198]. Together, above results suggest that pressor responses to noradrenaline microinjection into the BST are mediated by PVN magnocellular neurons without a significant involvement of SON neurons. They also suggest that local glutamatergic neurotransmission within the PVN through activation of non-NMDA glutamate receptors mediates this response. However, only a minor part of PVN-projecting neurons in the BST are glutamatergic. Indeed, as aforementioned, the vast majority of BNST projections to the PVN are GABAergic [74, 199]. Therefore, further experiments are necessary for a complete definition of neural pathway from the BNST to PVN involved in control of vasopressinergic magnocellular neurons. 

Recent results have suggested that noradrenergic and cholinergic mechanisms within the BNST modulate vasopressin release through different neural pathways. Alves and collaborators (2011) demonstrated that blockade of SON synapses, either ipsilateral or contralateral in relation to BST microinjection site, inhibited carbachol pressor response [200]. However, PVN ablation did not affect the carbachol-evoked pressor response [200]. These results suggested that carbachol cardiovascular changes are mediated by a neural pathway that depends on the activation of both ipsilateral and contralateral SON. This modulation of the vasopressin release by BST noradrenergic and cholinergic neuro-transmission through specific neural pathway may have a physiological importance, since BST neurons can stimulate vasopressin release through local cholinergic receptor despite changes in pathway related to local noradrenergic neurotransmission, or vice versa. A schematic representation sketching the mechanism by which noradrenaline and carbachol microinjection into the BST evokes vasopressin-mediated pressor response is presented in Fig. (**5**). Information regarding the neural pathway involved in control of vasopressin release by BNST GABAergic neurotransmission is missing.

It has been proposed that the BST is an important relay in the neural circuitry connecting limbic structures, such as the hippocampus, MPFC, MeA and CeA, to hypothalamic regions involved in neuroendocrine control [20, 48, 201]. However, a limited number of studies have investigated a possible involvement of the BNST in neural pathways involved in control of vasopressin release. In this way, it was reported that noradrenaline microinjection into the lateral septal area (LSA) evoked vasopressin-mediated pressor responses [202]. Moreover, similar to effects of noradrenaline microinjected into the BNST, pressor response to noradrenaline into the LSA were blocked by blockade of non-NMDA glutamate receptors in the PVN and not affected by blockade of local NMDA receptors [203]. Considering that the LSA does not project directly to PVN [204], and connections have been described between the BST and this hypothalamic structure [14], the BST could be a relay in the pathway of vasopressin release by the LSA. However, although noradrenaline microinjection into the LSA activate local BNST neurons [205], functional findings demonstrated that BNST inhibition did not affect cardiovascular responses to noradrenaline into the LSA [206]. Since other forebrain structures connected with BNST, such as MPFC and MeA, also modulates cardiovascular function through control of vasopressin release [197, 207], further studies are necessary to clarify the neural circuitry that the BNST is part. 

## 4 CONCLUSIONS AND FUTURE DIRECTIONS

Although the BNST is not involved in tonic maintenance of cardiovascular function under resting conditions, numerous results have demonstrated that it plays a key physiological role in integration of cardiovascular responses elicited by peripheral stimuli (e.g., baroreflex) as well as in autonomic and neuroendocrine adjustments observed during aversive threat and physical exercise. Interestingly, recent data suggest that cardiovascular regulation by some local neurotransmitters (noradrenaline, GABA and acetylcholine) are mediated by neuroendocrine mechanisms (vasopressin release into the circulation), thus suggesting a coordinated control of cardiovascular and neuroendocrine function by the BNST. Several local neurotransmitters mediate the control of autonomic and neuroendocrine function by the BNST. The innervation of these neurochemical mechanisms to different regions of the BNST is not homogeneous and provides a regionalization in control of physiological functions by this structure. Indeed, the anterior division is the critical BNST region involved in autonomic control. In contrast, the control of neuroendocrine function, specially the HPA axis, is regulated by the entire rostrocaudal extension of the BNST, but anterior and posterior division may have different roles.

The results discussed in the present review situates the BNST as a key component in the integration of physiological and behavioral responses, connecting limbic forebrain structures to hypothalamic and brainstem regions associated with autonomic and neuroendocrine functions.However, the role of local neurochemical mechanisms, especially in control of physiological responses to stress and physical exercise, is not completely understood. Furthermore, neurochemical and electrophysiological studies have demonstrated an interaction in action of neurotransmitters within the BNST, mainly through a modulation of presynaptic neurotransmitter release [70, 73, 99, 208, 209]. However, the physiological and physiopathological relevance of the complex interaction between neurochemical mechanisms in BNST control of cardiovascular and neuroendocrine function is not completely understood. Changes in BNST functioning may be part of neurogenic mechanisms involved in physiopathology of cardiovascular, neuroendocrine and psychiatric pathologies. Although some clinical and preclinical results provide initial evidence supporting this idea [32, 33], further studies with special focus on role of local neuropharmacological mechanisms are necessary.

## Figures and Tables

**Fig. (1):**
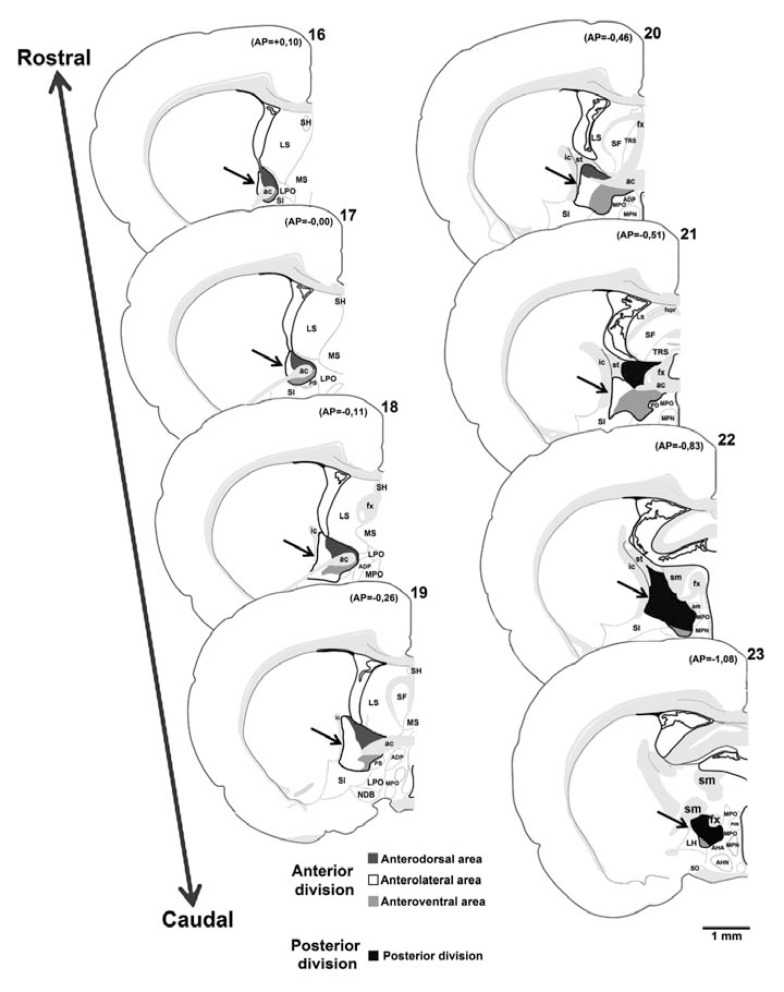
Schematic diagrams based on rat brain atlas of Swanson (2004) illustrating the BNST and its subregions. The atlas templates arranged from rostral (atlas level 16) to caudal (atlas level 23) level. The arrows indicate the BNST. The dark gray region show the anterodorsal area, the white region show the anterolateral area, the light gray region show the anteroventral area and the black region show the posterior division. ac, anterior commissure; ADP, anterodorsal preoptic nucleus; AHA, anterior hypothalamic area; AHN, anterior hypothalamic nucleus; am, amygdalar capsule; AP, antero-posterior (distance in mm from bregma); fx, fornix; ic, internal capsule; LH, lateral hypothalamic area; LPO, lateral preoptic area; LS, lateral septal nucleus; MPN, medial preoptic nucleus; MPO, medial preoptic area; MS, medial septal nucleus; NDB, diagonal band nucleus; PD, posterodorsal preoptic nucleus; PS, parastrial nucleus; PVH, paraventricular hypothalamic nucleus; SF, septofimbrial nucleus; SH, septohippocampal nucleus; SI, substance innominata; sm, stria medullaris; SO, supraoptic nucleus; st, stria terminalis; TRS, triangular nucleus septum.

**Fig. (5):**
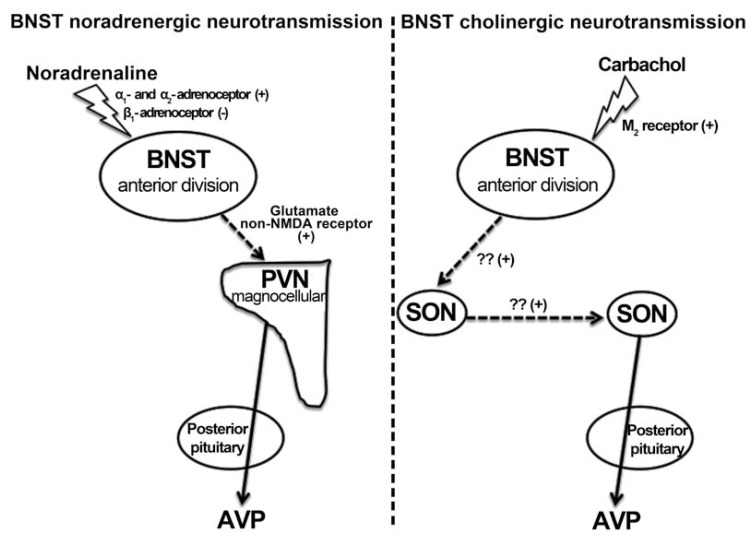
Schematic representation illustrating the neural pathways and neurochemical mechanisms by which noradrenaline and carbachol microinjection into the BNST evokes vasopressin release. AVP, arginine vasopressin; Glu, glutamate; M_2_ receptor, subtype M_2_ of muscarinic cholinergic receptors; PVN, paraventricular hypothalamic nucleus; SO, supraoptic hypothalamic nucleus.

**Table 1.** Summary of Main Findings and Mechanisms Associated with BNST Control of Cardiovascular Function

Animal Model	Treatment (Dose)	Local Mechanism within the BNST	Effects (Peripheral Mechanism)	Reference
**Cardiovascular responses**				
	Noradrenaline (10nmol)	- Co-activation of α_1_- and α_2_-adrenoceptor - Negatively modulated by β_1_ -adrenoceptor	↑ blood pressure ↓ HR (vasopressin release into the circulation)	Crestani *et al*, 2007; Crestani *et al*, 2008.
	Glutamate (0.25 - 1 M)	- Co-ativation of NMDA and non-NMDA receptors	↓blood pressure ↓ HR (Decrease in vascular and cardiac sympathetic outflow)	Ciriello & Janssen, 1993*; Geslema *et al*, 1993; Hatam & Nasimi, 2007*
	Bicuculline Methiodide (100 pmol)	GABA_A_ receptor antagonist	↑ blood pressure ↑ HR (vasopressin release into the circulation and cardiac sympathetic activation, respectively)	Hatam *et al*, 2009*
	Carbachol (0.1 - 3 nmol)	M2 receptor activation	↑ blood pressure ↓ HR (vasopressin release into the circulation)	Alves *et al*, 2007
	Acetylcholine (6 nmol)	Muscarinic receptor activation	↑ blood pressure none effect HR	Nasimi & Hatam, 2011*
	CRF (0.042 nmol)	CRF receptors agonist	↑ HR	Nijsen *et al*, 2001
**Baroreflex Modulation**				
	CoCl_2_ (1 nmol)	Synaptic blocker	Conscious animals ↑ reflex bradycardia none effect reflex tachycardia Anesthetized animals ↓ reflex bradycardia	Crestani *et al*., 2006; Nasimi and Hatam, 2011*
	WB4101 (15 nmol)	α_1_ - adrenoceptor antagonist	↑ reflex bradycardia (modulation of cardiac parasympathetic activity)	Crestani *et al*., 2008
	LY235959 (4 nmol)	NMDA receptor antagonist	↑ reflex bradycardia	Alves *et al*., 2009
	NPLA (0.04 nmol) Carboxy-PTIO (1 nmol)	nNOS inhibitor and NO scavenger, respectively	↑ reflex bradycardia	Alves *et al*., 2009
	Acetylcholine (6 nmol)	Muscarinic receptor activation	↓ reflex bradycardia	Nasimi and Hatam, 2011*
	Cannabidiol (60 nmol) 8-OH-DPAT (4 nmol)	5-HT_1A_ receptor activation	↑ reflex bradycardia	Alves *et al*., 2011
**Chemoreflex Modulation**				
	CoCl_2_ (1 nmol)	Synaptic blocker	None effect	Granjeiro *et al*., 2012
**Restraint stress**				
	CoCl_2_ (1 nmol)	Synaptic blocker	↑ tachycardiac response none effect pressor response	Crestani *et al*., 2009
	WB4101 (15 nmol)	α_1_ - adrenoceptor antagonist	None effect pressor response ↑ tachycardiac response (modulation of cardiac parasympathetic activity)	Crestani *et al*., 2009
	Cannabidiol (30 and 60 nmol)	5-HT1A receptor activation	↑ tachycardiac response none effect pressor response	Gomes *et al*., 2012
**Contextual fear conditioning**				
	α-helical CRF (9-41) (0.052 nmol)	CRF antagonist	↑ tachycardiac response (modulation of cardiac parasympathetic activity)	Nijsen *et al*., 2001
	CoCl_2_ (1 nmol)	Synaptic blocker	↓ pressor response ↓ tachycardiac response	Resstel *et al*., 2008
	WB4101 (1.7 nmol)	α_1_ - adrenoceptor antagonist	↓ pressor response ↓ tachycardiac response ↓ fall in tail cutaneous temperature	Hott *et al*., 2012
	CGP20712 (4.5 nmol)	β_1_ -adrenoceptor antagonist	↓ pressor response ↓ tachycardiac response ↓ fall in tail cutaneous temperature	Hott *et al*., 2012
	Cannabidiol (30 and 60 nmol)	5-HT1A receptor activation	↓ pressor response ↓ tachycardiac response	Gomes *et al*., 2012
**Dynamic exercise**				
	CoCl_2_ (1 nmol)	Synaptic blocker	↓ pressor response ↓ tachycardiac response	Crestani *et al*., 2010
	WB4101 (10 nmol)	α_1_ - adrenoceptor antagonist	↑ tachycardiac response None effect pressor response	Alves *et al*., 2011
	RX821002 (10 nmol)	α_2_ - adrenoceptor antagonist	↓ pressor response none effect tachycardiac response	Alves *et al*., 2011

* anesthetized animals.

↑: increase,↓: decrease, CRF: corticotropin-releasing factor,HR: heart rate, nNOS: neuronal nitric oxide synthase, NPLA: N-propyl-L-arginine.
